# Sixth Annual Meeting of the Mississippi Valley Association of Dental Surgeons

**Published:** 1850-10

**Authors:** James Taylor, Chas. Bonsall

**Affiliations:** President


### SIXTH ANNUAL MEETING OF THE MISSISSIPPI VALLEY ASSOCIATION OF DENTAL SURGEONS.

The Society met pursuant to adjournment at the Ohio College of Dental Surgery, Sept. 10th, 1850.

The Society came to order at 10 A. M., the President, Dr. James Taylor in the chair. The recording secretary being absent, on motion of Dr. Leslie, Dr. C. Bonsall was appointed secretary, pro tem. On calling the roll, the following members were found to be present, viz.: Drs. James Taylor, Bonsall, Berry, Ulrey, Griffith, Leslie, Tait, Curtiss, Van Emon, McCullom and Gray.

The President's address being first in order, he read an interesting paper on Plugging Teeth, which was, on motion, accepted and ordered to be published.

A letter was now received from Dr. W. M. Hunter, enclosing his dues and resigning his membership, which was accepted.

On motion, the Society adjourned to meet at 3 P. M.

### AFTERNOON SESSION.

The Society came to order at 3 P. M., when a discussion took place on the subject of Dr. Taylor's address, which continued with much interest most of the afternoon.

The examining committee having presented the name of Dr. J. W. Baxter, of Warsaw, Ky., the Society went into an election by ballot, when he was found to be duly elected a member.

On motion, adjourned to meet at the house of Dr. Bonsall, at 7½ P. M.

EVENING SESSION.

The Society met agreeably to adjournment, at Dr. Bonsall's. The President being absent, Dr. Griffith, Vice President, took the chair. Some time was spent in the further discussion of the subject of plugging teeth, after which, on motion, Dr. Bonsall's address on Alveolar Abscess being next in order, was read, accepted, and elicited considerable discussion.

Dr. Bonsall, appointed last year as a committee to have medals prepared and forwarded to Messrs. Jones, White & Co., and Mr. James Alcock for specimens of teeth, reported having attended to the duty, as he understood, to their satisfaction.

On motion, Drs. Taft, Leslie and Curtiss were appointed to examine the Treasurer's accounts.

On motion, adjourned, to meet to-morrow at 8 o'clock, A. M.

SECOND DAY—8 O'CLOCK A. M.

The Society met according to adjournment in the Dental College, when the minutes of yesterday's proceedings were read and adopted.

The committee to audit the Treasurer's accounts, handed in the following report which was accepted:

To the Mississippi Valley Association of Dental Surgeons:—

Your committee beg leave to report that they have examined the Treasurer's accounts, and find them correct, leaving a balance of $88.60 in his hands.

J. TAFT,<br>

A. M. LESLIE, } *Committee.*<br>

J. G. CURTISS.

Dr. Van Emon's address not being ready, he was requested to have it prepared by next year.

Dr. Taft then read his address on Dentition which was accepted, and after discussion he was requested to continue the subject next year.

Dr. Gray then read his address, and after acceptance and discussion, he also was requested to continue the subject next year.

On motion, Drs. Berry, Gray and Taft were appointed a committee to audit the accounts of the Publishing Committee of the "Register."

On motion, adjourned till 3 P. M.

AFTERNOON SESSION.

The Society met according to adjournment.

The committee to audit the Publishing committee's accounts reported the following, which was accepted:

To the Mississippi Valley Association of Dental Surgeons:

*Gentlemen:* Your committee to audit the accounts of the Publishing committee would report that they have performed the duty, and find the expense of publishing the "Dental Register of the West," including the three volumes issued, to be $484.84, and the receipts from the same to be $442.00, leaving a balance of $42.84 due the Publishing committee, all which is respectfully submitted.

Cincinnati, Sept. 11, 1850.

A. Berry,

S. C. Gray,

J. Taft,

} *Committee.*

On motion of Dr. Berry, ten dollars was allowed to the Publishing committee for stationery, postage and extra charges for printing during the last year.

On motion, the Publishing committee was authorized to draw on the Treasurer for the amount now due them.

On motion, Drs. Berry and Goddard were added to the committee to answer Dr. Drake's enquiries.

On motion, the Society went into an election for officers for the ensuing year, when the following gentlemen were found to be elected:

Samuel Griffith, President,
A. Berry, 1st Vice do.
J. Taft, 2d do do.
J. P. Ulrey, 3d do do.
W. H. Goddard, Corresponding Secretary.
Jno. Allen, Recording Secretary.
Charles Bonsall, Treasurer.
W. H. Goddard,
Jno. Allen,
S. C. Gray,
} Examining Committee.
A. M. Leslie,
J. G. Curtiss,
G. L. Van Emon.
} Executive Committee.
James Taylor, Editor of the "Register."

The following gentlemen were appointed Essayists for the year 1851:

Dr. Taft on Dentition.

" Gray on the connection of Chemistry with Dentistry.

" Van Emon on the Blow Pipe.

" Baxter on the use of Palladium in Dentistry.

" Ulrey on Atmospheric pressure plates.

" Berry on Constitutional peculiarities considered as developed under dental operati ons.

On motion, adjourned, to meet at Dr. Leslie's office, at 7½ o'clock, P. M.

EVENING SESSION.

The Society met according to adjournment at Dr. Leslie's office; the minutes of the previous meeting were read and adopted.

Most of the evening was devoted to an interlocutory discussion on the subject of atmospheric pressure plates, at the end of which, it was on motion of Dr. Griffith,

*Resolved*, That we now adjourn, to meet in Louisville on the second Wednesday in September, 1851, at 10 A. M.

James Taylor, President.

Chas. Bonsall, Secretary, pro tem.

